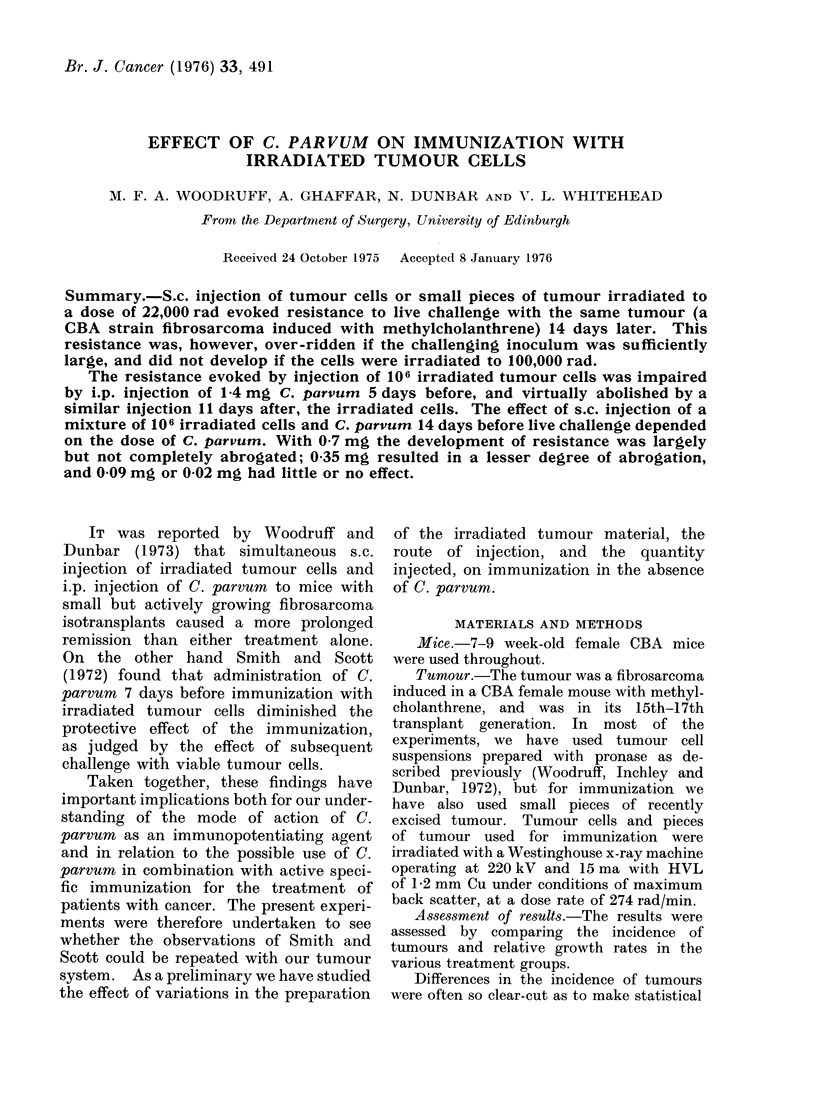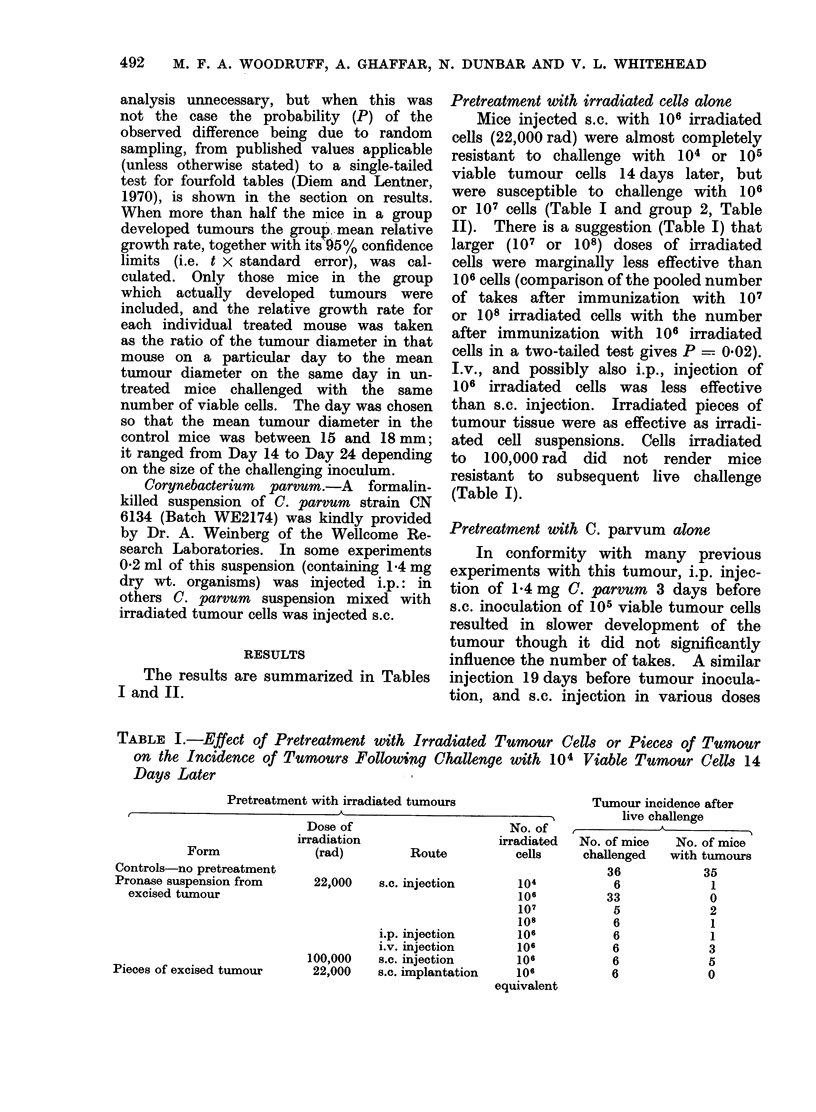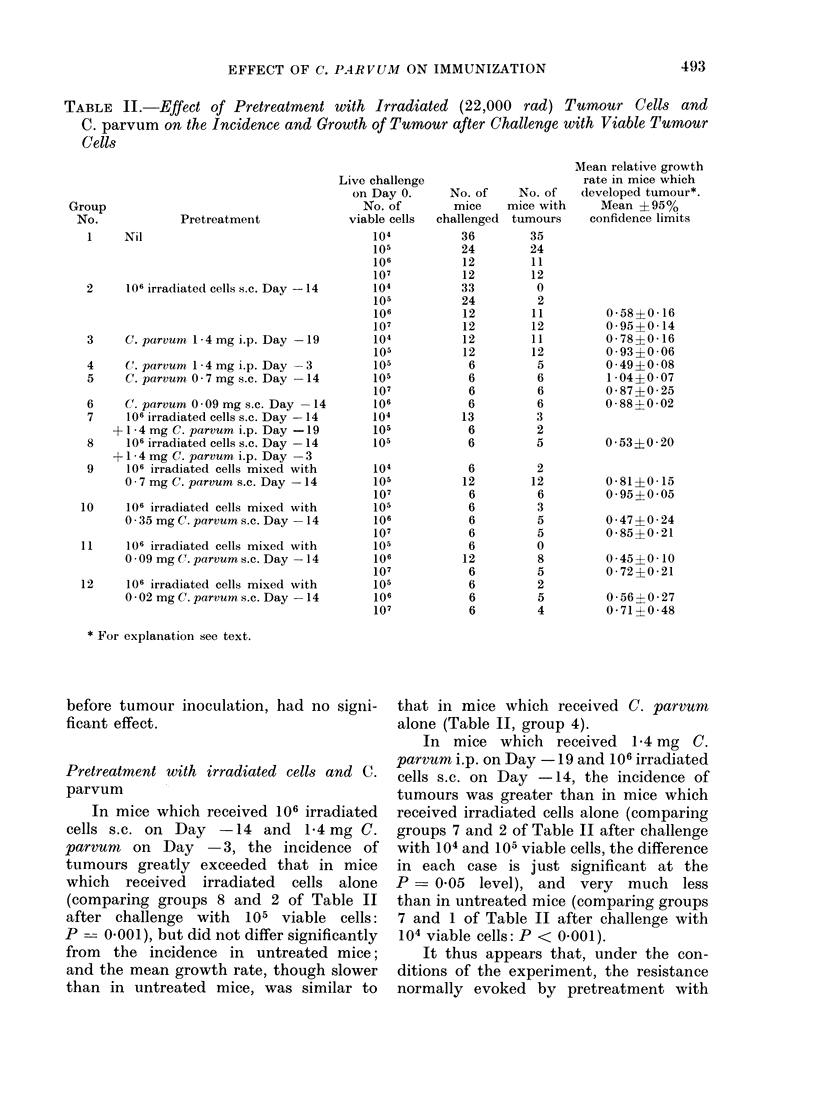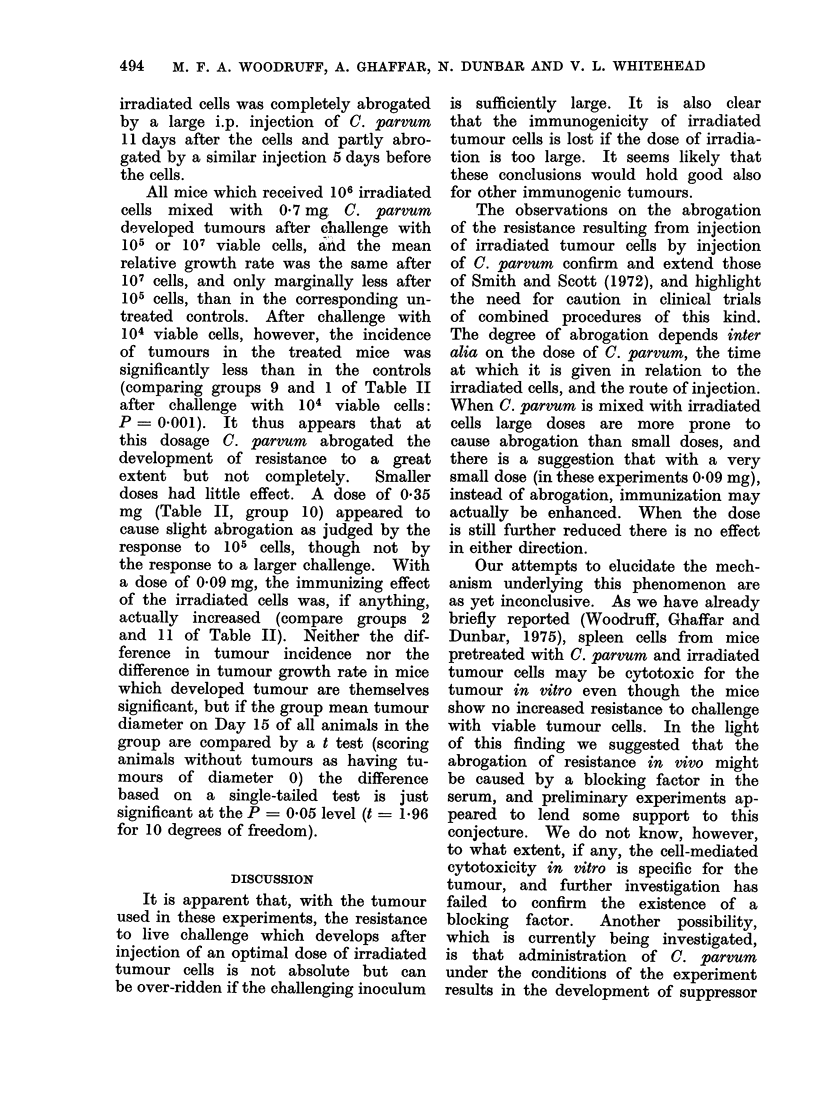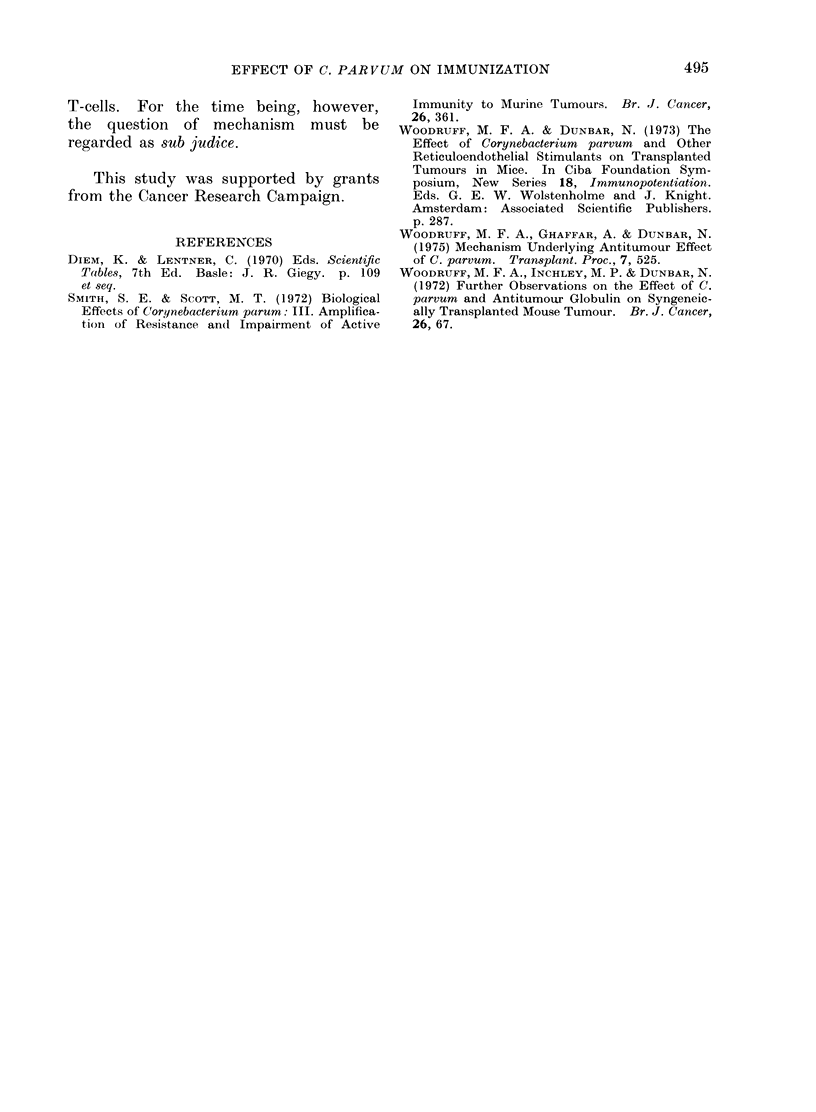# Effect of C. Parvum on immunization with irradiated tumour cells.

**DOI:** 10.1038/bjc.1976.79

**Published:** 1976-05

**Authors:** M. F. Woodruff, A. Ghaffar, N. Dunbar, V. L. Whitehead

## Abstract

S.c. injection of tumour cells or small pieces of tumour irradiated to a dose of 22,000 rad evoked resistance to live challenge with the same tumour (a CBA strain fibrosarcoma induced with methylcholanthrene) 14 days later. This resistance was, however, over-ridden if the challenging inoculum was sufficiently large, and did not develop if the cells were irradiated to 100,000 rad. The resistance evoked by injection of 10(6) irradiated tumour cells was impaired by i.p. injection of 1-4 mg C. parvum 5 days before, and virtually abolished by a similar injection 11 days after, the irradiated cells. The effect of s.c. injection of a mixture of 10(6) irradiated cells and C. parvum 14 days before live challenge depended on the dose of C. parvum. With 0-7 mg the development of resistance was largely but not completely abrogated; 0-35 mg resulted in a lesser degree of abrogation, and 0-09 mg or 0-02 mg had little or no effect.


*Br. J. Cancer* (1976) **33**, 491

### EFFECT OF *C. PARVUM* ON IMMUNIZATION WITH IRRADIATED TUMOUR CELLS

M. F. A. WOODRUFF, A. GHAFFAR, N. DUNBAR AND V. L. WHITEHEAD

*From the Department of Surgery, University of Edinburgh*

Received 24 October 1975 Accepted 8 January 1976

**Summary.—S.c. injection of tumour cells or small pieces of tumour irradiated to a dose of 22,000 rad evoked resistance to live challenge with the same tumour (a CBA strain fibrosarcoma induced with methylcholanthrene) 14 days later. This resistance was, however, over-ridden if the challenging inoculum was sufficiently large, and did not develop if the cells were irradiated to 100,000 rad.**

**The resistance evoked by injection of $10^6$ irradiated tumour cells was impaired by i.p. injection of 1·4 mg *C. parvum* 5 days before, and virtually abolished by a similar injection 11 days after, the irradiated cells. The effect of s.c. injection of a mixture of $10^6$ irradiated cells and *C. parvum* 14 days before live challenge depended on the dose of *C. parvum*. With 0·7 mg the development of resistance was largely but not completely abrogated; 0·35 mg resulted in a lesser degree of abrogation, and 0·09 mg or 0·02 mg had little or no effect.**

IT was reported by Woodruff and Dunbar (1973) that simultaneous s.c. injection of irradiated tumour cells and i.p. injection of *C. parvum* to mice with small but actively growing fibrosarcoma isotransplants caused a more prolonged remission than either treatment alone. On the other hand Smith and Scott (1972) found that administration of *C. parvum* 7 days before immunization with irradiated tumour cells diminished the protective effect of the immunization, as judged by the effect of subsequent challenge with viable tumour cells.

Taken together, these findings have important implications both for our understanding of the mode of action of *C. parvum* as an immunopotentiating agent and in relation to the possible use of *C. parvum* in combination with active specific immunization for the treatment of patients with cancer. The present experiments were therefore undertaken to see whether the observations of Smith and Scott could be repeated with our tumour system. As a preliminary we have studied the effect of variations in the preparation of the irradiated tumour material, the route of injection, and the quantity injected, on immunization in the absence of *C. parvum*.

### MATERIALS AND METHODS

*Mice.*—7–9 week-old female CBA mice were used throughout.

*Tumour.*—The tumour was a fibrosarcoma induced in a CBA female mouse with methylcholanthrene, and was in its 15th–17th transplant generation. In most of the experiments, we have used tumour cell suspensions prepared with pronase as described previously (Woodruff, Inchley and Dunbar, 1972), but for immunization we have also used small pieces of recently excised tumour. Tumour cells and pieces of tumour used for immunization were irradiated with a Westinghouse x-ray machine operating at 220 kV and 15 ma with HVL of 1·2 mm Cu under conditions of maximum back scatter, at a dose rate of 274 rad/min.

*Assessment of results.*—The results were assessed by comparing the incidence of tumours and relative growth rates in the various treatment groups.

Differences in the incidence of tumours were often so clear-cut as to make statistical analysis unnecessary, but when this was not the case the probability ($P$) of the observed difference being due to random sampling, from published values applicable (unless otherwise stated) to a single-tailed test for fourfold tables (Diem and Lentner, 1970), is shown in the section on results. When more than half the mice in a group developed tumours the group mean relative growth rate, together with its 95% confidence limits (i.e. $t \times$ standard error), was calculated. Only those mice in the group which actually developed tumours were included, and the relative growth rate for each individual treated mouse was taken as the ratio of the tumour diameter in that mouse on a particular day to the mean tumour diameter on the same day in untreated mice challenged with the same number of viable cells. The day was chosen so that the mean tumour diameter in the control mice was between 15 and 18 mm; it ranged from Day 14 to Day 24 depending on the size of the challenging inoculum.

*Corynebacterium parvum*.—A formalin-killed suspension of *C. parvum* strain CN 6134 (Batch WE2174) was kindly provided by Dr. A. Weinberg of the Wellcome Research Laboratories. In some experiments 0·2 ml of this suspension (containing 1·4 mg dry wt. organisms) was injected i.p.: in others *C. parvum* suspension mixed with irradiated tumour cells was injected s.c.

### RESULTS

The results are summarized in Tables I and II.

*Pretreatment with irradiated cells alone*

Mice injected s.c. with $10^6$ irradiated cells (22,000 rad) were almost completely resistant to challenge with $10^4$ or $10^5$ viable tumour cells 14 days later, but were susceptible to challenge with $10^6$ or $10^7$ cells (Table I and group 2, Table II). There is a suggestion (Table I) that larger ($10^7$ or $10^8$) doses of irradiated cells were marginally less effective than $10^6$ cells (comparison of the pooled number of takes after immunization with $10^7$ or $10^8$ irradiated cells with the number after immunization with $10^6$ irradiated cells in a two-tailed test gives $P = 0{\cdot}02$). I.v., and possibly also i.p., injection of $10^6$ irradiated cells was less effective than s.c. injection. Irradiated pieces of tumour tissue were as effective as irradiated cell suspensions. Cells irradiated to 100,000 rad did not render mice resistant to subsequent live challenge (Table I).

*Pretreatment with* C. parvum *alone*

In conformity with many previous experiments with this tumour, i.p. injection of 1·4 mg *C. parvum* 3 days before s.c. inoculation of $10^5$ viable tumour cells resulted in slower development of the tumour though it did not significantly influence the number of takes. A similar injection 19 days before tumour inoculation, and s.c. injection in various doses

TABLE I.—*Effect of Pretreatment with Irradiated Tumour Cells or Pieces of Tumour on the Incidence of Tumours Following Challenge with* $10^4$ *Viable Tumour Cells 14 Days Later*

| Pretreatment with irradiated tumours | | | | Tumour incidence after live challenge | |
|---|---|---|---|---|---|
| Form | Dose of irradiation (rad) | Route | No. of irradiated cells | No. of mice challenged | No. of mice with tumours |
| Controls—no pretreatment | | | | 36 | 35 |
| Pronase suspension from excised tumour | 22,000 | s.c. injection | $10^4$ | 6 | 1 |
| | | | $10^6$ | 33 | 0 |
| | | | $10^7$ | 5 | 2 |
| | | | $10^8$ | 6 | 1 |
| | | i.p. injection | $10^6$ | 6 | 1 |
| | | i.v. injection | $10^6$ | 6 | 3 |
| | 100,000 | s.c. injection | $10^6$ | 6 | 5 |
| Pieces of excised tumour | 22,000 | s.c. implantation | $10^6$ equivalent | 6 | 0 |

TABLE II.—*Effect of Pretreatment with Irradiated* (22,000 *rad*) *Tumour Cells and* C. parvum *on the Incidence and Growth of Tumour after Challenge with Viable Tumour Cells*

| Group No. | Pretreatment | Live challenge on Day 0. No. of viable cells | No. of mice challenged | No. of mice with tumours | Mean relative growth rate in mice which developed tumour*. Mean ±95% confidence limits |
|---|---|---|---|---|---|
| 1 | Nil | $10^4$ | 36 | 35 | |
| | | $10^5$ | 24 | 24 | |
| | | $10^6$ | 12 | 11 | |
| | | $10^7$ | 12 | 12 | |
| 2 | $10^6$ irradiated cells s.c. Day −14 | $10^4$ | 33 | 0 | |
| | | $10^5$ | 24 | 2 | |
| | | $10^6$ | 12 | 11 | $0·58 \pm 0·16$ |
| | | $10^7$ | 12 | 12 | $0·95 \pm 0·14$ |
| 3 | *C. parvum* 1·4 mg i.p. Day −19 | $10^4$ | 12 | 11 | $0·78 \pm 0·16$ |
| | | $10^5$ | 12 | 12 | $0·93 \pm 0·06$ |
| 4 | *C. parvum* 1·4 mg i.p. Day −3 | $10^5$ | 6 | 5 | $0·49 \pm 0·08$ |
| 5 | *C. parvum* 0·7 mg s.c. Day −14 | $10^5$ | 6 | 6 | $1·04 \pm 0·07$ |
| | | $10^7$ | 6 | 6 | $0·87 \pm 0·25$ |
| 6 | *C. parvum* 0·09 mg s.c. Day −14 | $10^6$ | 6 | 6 | $0·88 \pm 0·02$ |
| 7 | $10^6$ irradiated cells s.c. Day −14 | $10^4$ | 13 | 3 | |
| | +1·4 mg *C. parvum* i.p. Day −19 | $10^5$ | 6 | 2 | |
| 8 | $10^6$ irradiated cells s.c. Day −14 | $10^5$ | 6 | 5 | $0·53 \pm 0·20$ |
| | +1·4 mg *C. parvum* i.p. Day −3 | | | | |
| 9 | $10^6$ irradiated cells mixed with | $10^4$ | 6 | 2 | |
| | 0·7 mg *C. parvum* s.c. Day −14 | $10^5$ | 12 | 12 | $0·81 \pm 0·15$ |
| | | $10^7$ | 6 | 6 | $0·95 \pm 0·05$ |
| 10 | $10^6$ irradiated cells mixed with | $10^5$ | 6 | 3 | |
| | 0·35 mg *C. parvum* s.c. Day −14 | $10^6$ | 6 | 5 | $0·47 \pm 0·24$ |
| | | $10^7$ | 6 | 5 | $0·85 \pm 0·21$ |
| 11 | $10^6$ irradiated cells mixed with | $10^5$ | 6 | 0 | |
| | 0·09 mg *C. parvum* s.c. Day −14 | $10^6$ | 12 | 8 | $0·45 \pm 0·10$ |
| | | $10^7$ | 6 | 5 | $0·72 \pm 0·21$ |
| 12 | $10^6$ irradiated cells mixed with | $10^5$ | 6 | 2 | |
| | 0·02 mg *C. parvum* s.c. Day −14 | $10^6$ | 6 | 5 | $0·56 \pm 0·27$ |
| | | $10^7$ | 6 | 4 | $0·71 \pm 0·48$ |

* For explanation see text.

before tumour inoculation, had no significant effect.

### *Pretreatment with irradiated cells and* C. parvum

In mice which received $10^6$ irradiated cells s.c. on Day −14 and 1·4 mg *C. parvum* on Day −3, the incidence of tumours greatly exceeded that in mice which received irradiated cells alone (comparing groups 8 and 2 of Table II after challenge with $10^5$ viable cells: $P = 0·001$), but did not differ significantly from the incidence in untreated mice; and the mean growth rate, though slower than in untreated mice, was similar to that in mice which received *C. parvum* alone (Table II, group 4).

In mice which received 1·4 mg *C. parvum* i.p. on Day −19 and $10^6$ irradiated cells s.c. on Day −14, the incidence of tumours was greater than in mice which received irradiated cells alone (comparing groups 7 and 2 of Table II after challenge with $10^4$ and $10^5$ viable cells, the difference in each case is just significant at the $P = 0·05$ level), and very much less than in untreated mice (comparing groups 7 and 1 of Table II after challenge with $10^4$ viable cells: $P < 0·001$).

It thus appears that, under the conditions of the experiment, the resistance normally evoked by pretreatment with irradiated cells was completely abrogated by a large i.p. injection of *C. parvum* 11 days after the cells and partly abrogated by a similar injection 5 days before the cells.

All mice which received $10^6$ irradiated cells mixed with 0·7 mg *C. parvum* developed tumours after challenge with $10^5$ or $10^7$ viable cells, and the mean relative growth rate was the same after $10^7$ cells, and only marginally less after $10^5$ cells, than in the corresponding untreated controls. After challenge with $10^4$ viable cells, however, the incidence of tumours in the treated mice was significantly less than in the controls (comparing groups 9 and 1 of Table II after challenge with $10^4$ viable cells: $P = 0{\cdot}001$). It thus appears that at this dosage *C. parvum* abrogated the development of resistance to a great extent but not completely. Smaller doses had little effect. A dose of 0·35 mg (Table II, group 10) appeared to cause slight abrogation as judged by the response to $10^5$ cells, though not by the response to a larger challenge. With a dose of 0·09 mg, the immunizing effect of the irradiated cells was, if anything, actually increased (compare groups 2 and 11 of Table II). Neither the difference in tumour incidence nor the difference in tumour growth rate in mice which developed tumour are themselves significant, but if the group mean tumour diameter on Day 15 of all animals in the group are compared by a $t$ test (scoring animals without tumours as having tumours of diameter 0) the difference based on a single-tailed test is just significant at the $P = 0{\cdot}05$ level ($t = 1{\cdot}96$ for 10 degrees of freedom).

### DISCUSSION

It is apparent that, with the tumour used in these experiments, the resistance to live challenge which develops after injection of an optimal dose of irradiated tumour cells is not absolute but can be over-ridden if the challenging inoculum is sufficiently large. It is also clear that the immunogenicity of irradiated tumour cells is lost if the dose of irradiation is too large. It seems likely that these conclusions would hold good also for other immunogenic tumours.

The observations on the abrogation of the resistance resulting from injection of irradiated tumour cells by injection of *C. parvum* confirm and extend those of Smith and Scott (1972), and highlight the need for caution in clinical trials of combined procedures of this kind. The degree of abrogation depends *inter alia* on the dose of *C. parvum*, the time at which it is given in relation to the irradiated cells, and the route of injection. When *C. parvum* is mixed with irradiated cells large doses are more prone to cause abrogation than small doses, and there is a suggestion that with a very small dose (in these experiments 0·09 mg), instead of abrogation, immunization may actually be enhanced. When the dose is still further reduced there is no effect in either direction.

Our attempts to elucidate the mechanism underlying this phenomenon are as yet inconclusive. As we have already briefly reported (Woodruff, Ghaffar and Dunbar, 1975), spleen cells from mice pretreated with *C. parvum* and irradiated tumour cells may be cytotoxic for the tumour *in vitro* even though the mice show no increased resistance to challenge with viable tumour cells. In the light of this finding we suggested that the abrogation of resistance *in vivo* might be caused by a blocking factor in the serum, and preliminary experiments appeared to lend some support to this conjecture. We do not know, however, to what extent, if any, the cell-mediated cytotoxicity *in vitro* is specific for the tumour, and further investigation has failed to confirm the existence of a blocking factor. Another possibility, which is currently being investigated, is that administration of *C. parvum* under the conditions of the experiment results in the development of suppressor

T-cells. For the time being, however, the question of mechanism must be regarded as *sub judice*.

This study was supported by grants from the Cancer Research Campaign.

### REFERENCES

DIEM, K. & LENTNER, C. (1970) Eds. *Scientific Tables*, 7th Ed. Basle: J. R. Giegy. p. 109 *et seq*.

SMITH, S. E. & SCOTT, M. T. (1972) Biological Effects of *Corynebacterium parum*: III. Amplification of Resistance and Impairment of Active Immunity to Murine Tumours. *Br. J. Cancer*, **26**, 361.

WOODRUFF, M. F. A. & DUNBAR, N. (1973) The Effect of *Corynebacterium parvum* and Other Reticuloendothelial Stimulants on Transplanted Tumours in Mice. In Ciba Foundation Symposium, New Series **18**, *Immunopotentiation*. Eds. G. E. W. Wolstenholme and J. Knight. Amsterdam: Associated Scientific Publishers. p. 287.

WOODRUFF, M. F. A., GHAFFAR, A. & DUNBAR, N. (1975) Mechanism Underlying Antitumour Effect of *C. parvum*. *Transplant. Proc.*, **7**, 525.

WOODRUFF, M. F. A., INCHLEY, M. P. & DUNBAR, N. (1972) Further Observations on the Effect of *C. parvum* and Antitumour Globulin on Syngeneically Transplanted Mouse Tumour. *Br. J. Cancer*, **26**, 67.